# Potential Probiotic *Lacticaseibacillus paracasei* MJM60396 Prevents Hyperuricemia in a Multiple Way by Absorbing Purine, Suppressing Xanthine Oxidase and Regulating Urate Excretion in Mice

**DOI:** 10.3390/microorganisms10050851

**Published:** 2022-04-20

**Authors:** Youjin Lee, Pia Werlinger, Joo-Won Suh, Jinhua Cheng

**Affiliations:** 1 Graduate School of Interdisciplinary Program of Biomodulation, Myongji University, Yongin 17058, Korea; youjin247@naver.com (Y.L.); pia.werlinger@gmail.com (P.W.); 2 Myongji Bioefficacy Research Center, Myongji University, Yongin 17058, Korea; jwsuh@mju.ac.kr

**Keywords:** probiotics, *L. paracasei* MJM60396, hyperuricemia, purine degradation, uric acid, xanthine oxidase, tight junction protein, microbiota

## Abstract

Hyperuricemia is a metabolic disorder caused by increased uric acid (UA) synthesis or decreased UA excretion. Changes in eating habits have led to an increase in the consumption of purine-rich foods, which is closely related to hyperuricemia. Therefore, decreased purine absorption, increased UA excretion, and decreased UA synthesis are the main strategies to ameliorate hyperuricemia. This study aimed to screen the lactic acid bacteria (LAB) with purine degrading ability and examine the serum UA-lowering effect in a hyperuricemia mouse model. As a result, *Lacticaseibacillus paracasei* MJM60396 was selected from 22 LAB isolated from fermented foods for 100% assimilation of inosine and guanosine. MJM60396 showed probiotic characteristics and safety properties. In the animal study, the serum uric acid was significantly reduced to a normal level after oral administration of MJM60396 for 3 weeks. The amount of xanthine oxidase, which catalyzes the formation of uric acid, decreased by 81%, and the transporters for excretion of urate were upregulated. Histopathological analysis showed that the damaged glomerulus, Bowman’s capsule, and tubules of the kidney caused by hyperuricemia was relieved. In addition, the impaired intestinal barrier was recovered and the expression of tight junction proteins, ZO-1 and occludin, was increased. Analysis of the microbiome showed that the relative abundance of *Muribaculaceae* and *Lachnospiraceae* bacteria, which were related to the intestinal barrier integrity, was increased in the MJM60396 group. Therefore, these results demonstrated that *L. paracasei* MJM60396 can prevent hyperuricemia in multiple ways by absorbing purines, decreasing UA synthesis by suppressing xanthine oxidase, and increasing UA excretion by regulating urate transporters.

## 1. Introduction

Lifestyle changes, such as increased consumption of meat and processed foods, have increased the incidence of hyperuricemia and caused health problems worldwide [1]. Generally, uric acid (UA) in human plasma ranges from 4 to 7 mg/dL, and above or below this range is considered abnormal and generally referred to as hyperuricemia or hypouricemia, respectively [2]. During primate evolution, urate oxidase was lost from hominoids [3]. Thus, elevated serum levels of uric acid, the final metabolite of purines, causes hyperuricemia and gout [4,5]. Conditions with elevated blood uric acid levels have been reported to be associated with many metabolic disorders. However, hyperuricemia is commonly asymptomatic. It is not recommended to prescribe drugs to reduce the uric acid levels for hyperuricemia patients without symptoms. Therefore, it is very important to find a safe and alternative way to manage hyperuricemia.

Probiotics, known as “live microorganisms that, when administered in adequate amounts, confer a health benefit on the host”, are generally considered safe, and long-term use is well tolerated [6]. In many studies, effects such as maintaining normal intestinal microbiota [7], immunomodulatory activity [8], hypocholesterolemia effect [9], hypotension activity [10], anticancer activity [11], and prevention of diarrhea [12] have been demonstrated to be related to the regulation of the strain itself and the gut microbiota [13,14]. Previous studies showed that a probiotic strain, *Lactobacillus gasseri* PA3, was able to absorb purines, thereby reducing intestinal absorption in the body and lowering serum uric acid levels [15,16,17].

Xanthine oxidase (XO) is the key enzyme in the production of uric acid. In the human body, the endogenous and exogenous nucleotides are converted to nucleosides, then to purine bases. The corresponding degradation products are guanine from guanosine and hypoxanthine from inosine, which were converted from adenosine by adenosine deaminase. The guanine is metabolized to xanthine by guanine deaminase, then to uric acid by XO. Hypoxanthine is converted to xanthine, then to uric acid by XO [18]. Therefore, the increased XO activity can lead to increased serum uric acid, the final metabolite of purine degradation.

In humans, the excretion of urate mainly occurs in the kidney and intestine: 70% of the uric acid in the body is filtered out by the kidneys and excreted in the urine [19]. The remaining 30% is excreted in the intestine [20,21]. Several studies have shown that uric acid is a potential cause of deterioration of kidney function. Elevated uric acid levels have been shown to alter the basic structure of renal histology and have been implicated in both acute and chronic renal failure [22]. Hyperuricemia makes symptoms worse in people with colon cancer [23]. Intestinal barrier dysfunction and subsequent enhanced intestinal permeability may occur as a result of hyperuricemia [24]. About 30% of UA has been eliminated in the intestine via urate transporters [25]. The intestinal epithelium and the apical junction complex constitute the intestinal barrier, a dynamic interface between the internal and external environment that plays an important role in maintaining intestinal homeostasis [26]. ZO-1 and occludin are considered to be two of the primary tight junction proteins [27]. It is reported that hyperuricemia increases intestinal permeability and affects the diversity and abundance of the gut microbiome [28,29].

In the present study, we screened and isolated a candidate strain with high purine-degrading activity, and the anti-hyperuricemia activity was assessed in potassium oxonate-induced hyperuricemia mice supplemented with a high purine diet. The possible mechanism for lowering serum uric acid level was also investigated by purine absorption, uric acid synthesis, and metabolism. Furthermore, the capability of passing through the stimulated oro-gastro-intestinal digestive system, and the ability to adhere to the intestinal epithelium and colon were examined for the development of effective probiotics [30]. For safety tests, hemolytic activity, D-lactate production, bile salt deconjugation, biogenic amine production, and antibiotic susceptibility were determined.

## 2. Materials and Methods

### 2.1. Screening of Purine Degrading Lactobacillus Strains

#### 2.1.1. Incubation of Lactic Acid Bacteria (LAB) in Nucleoside Solution

A total 22 of LAB strains isolated from fermentation food were tested for their purine degrading ability. The *Lactobacillus* strains were inoculated into de Man, Rogosa, and Sharpe (MRS) medium (Difco, Seoul, Korea) and cultured in anaerobiosis at 37 °C for 48 h. After incubation, 2 mL of culture broth was centrifuged at 4000× *g* for 10 min. After washing three times with 1 mL of 0.85% NaCl, the cells were resuspended in 750 µL of 1 mM inosine-1 mM guanosine solution and incubated at 37 °C for 120 min. After that, the solution was centrifuged at 4000× *g* for 10 min. Next, 270 µL of the supernatant was taken from the supernatant, and 30 µL of 0.1 M HClO_4_ solution was added to stop the reaction. The residual inosine and guanosine in the solution were determined by using a high-performance liquid chromatography (HPLC) system as below.

#### 2.1.2. HPLC Analysis

HPLC analysis was performed by using a YMC ODS column (S-5 um, 12 mm, 250 × 4.6 mm) connected to a binary HPLC pump (Waters 1525), and the column temperature remained at 37 °C. The isocratic elution was performed with a NaClO_4_-H_3_PO_4_ solution (NaClO_4,_ 0.1 mM; H_3_PO_4,_ 0.187 M in dH_2_O), at a flow rate of 1 mL/min. Inosine, guanosine, and adenosine were detected at 254 nm by retention times of 21.8, 17.3, and 11.8 min respectively, and were quantified by comparing with the standard. The degrading speed and rate of inosine or guanosine by different LAB strains were calculated according to the following formula: V = (C − X)/120, D = [(C − X)/C] × 100%, where V is degradation speed (g/L/min); C denotes content of inosine or guanosine (g/L); X represents the residual content of inosine or guanosine (g/L); and D indicates the degradation rate (%).

#### 2.1.3. Degradation of Purine Compounds by the Cell Lysates of MJM60396 and MJM60662

Degradation of nucleoside was investigated by live cells and cell lysates. LAB strains were inoculated in MRS broth and cultured for 48 h at 37 °C under anaerobic conditions. Then 2 mL of the culture broth was centrifuged at 4000× *g*, 4 °C for 10 min. The cells were then washed twice with 1 mL of 0.85% NaCl and used as live cells. To make a lysis pellet and lysis supernatant, the cells were treated in 3 mL of 1 mM lysozyme solution for 3 h, and then the cells were collected and freeze-dried. The freeze-dried LAB was weighed and resuspended with 0.85% NaCl at 10 mg/mL followed by sonication. The cells were centrifuged at 1500× *g* for 10 min, yielding the lysis supernatant containing soluble material such as cell cytosol and the lysis pellet containing the cell membrane [31].

The live cell, lysis pellet, and lysis supernatant were resuspended with 750 µL of inosine, guanosine, and adenosine solution and incubated at 37 °C for 120 min, with shaking (120 rpm). After that, the solution was centrifuged at 4000× *g*, 4 °C for 10 min, and 270 µL of the supernatant was taken and mixed thoroughly with 30 µL of 0.1 M HClO_4_ to prevent further degradation. Then 20 µL of the mixture was injected into the HPLC device after filtration. The degradation rate of inosine and guanosine were calculated by the formula mentioned above.

### 2.2. Strain Identification by 16S rDNA Sequence and Phylogenetic Analysis of MJM60396

To identify the LAB isolated from fermented food, 16Sr DNA sequence was used as a marker gene to identify the species. Briefly, genomic DNA was isolated using Exgene cell SV DNA isolation kit (GeneAll, Seoul, Korea). The 16S rDNA sequence was amplified by using universal primer 27F, and 1492R, and sequenced. The resulted sequence was blasted in the Ezbiocloud database (ChunLab Inc., Seoul, Korea).

For phylogenetic analysis of MJM60396, the 16S rDNA sequence of closely related strains was aligned, and a phylogenetic tree was constructed with the neighbor-joining method using MEGA X software [32].

### 2.3. Utilization of Purine Compounds by MJM60396, MJM60662

To test the utilization of purine and nucleoside by MJM60396, a chemically defined growth (DM) medium [15] supplemented with purine nucleotide or nucleoside or base was used for the utilization assay. DM medium containing different purine types (final concentration of 400 µM) were inoculated with LAB at a concentration of 2 × 10^8^ CFU/mL and incubated at 37 °C for 0, 2, 4, 6, and 8 h under anaerobic conditions. The growth of LAB was monitored at OD_600_ by using a spectrophotometer (Tecan, infinite M200PRO, Grödig, Austria). DM medium without any purine or nucleoside was inoculated with the same amount of LAB and used as a control. All experiments were repeated 3 times.

### 2.4. Animal Study

#### 2.4.1. Animal Monitoring and Treatment

Male C57BL/6 mice (7 weeks old, *n* = 8 per group) were purchased from Raon Bio (Gyeonggi, Korea). All animals were housed in standard plastic cages and maintained under a 12-h light-dark cycle at constant temperature and humidity (22 ± 2 °C and 55 ± 5%, respectively) with free access to food and water. The protocols for the animal experiment were approved by the Committee on the Ethics of Animal Experiments of the Myongji Bioefficacy Research Center in Myongji University (MJIACUC-2021001), and the mice were maintained following the NIH guidelines for the care and use of laboratory animals.

After acclimatization for 1 week, the mice were divided into five groups: (1) normal diet control group (ND), (2) hyperuricemia group (HU), (3) allopurinol treated group (ALLO), (4) MJM60662 treated group (MJM60662 group) and (5) MJM60396 treated group (MJM60396 group). Rodents create an enzyme called uricase that breaks down uric acid, which reduces uric acid levels in the serum. To suppress the activity of uricase, potassium oxonate (300 mg/kg B.W. per day)-carboxymethylcellulose sodium (GMC-Na) solution (3 g/L) was intraperitoneally injected into groups 2 to 5 daily. Meanwhile, groups 2 to 5 were supplied with a high purine diet (HPD) containing an additional 570 g yeast extract in the normal diet (Raon Bio, Gyeonggi, Korea), while the ND group was supplied with a normal diet.

For treatment, LAB was cultured freshly daily, washed, and resuspended in saline (0.85% NaCl) for use. The MJM60662 group and MJM60396 group were administered *L.* *gasseri* MJM60662 (3 × 10^9^ CFU/mice/day) and *Lacticaseibacillus* *paracasei* MJM60396 (3 × 10^9^ CFU/mice/day) once daily for 21 days by oral gavage, and the control ND group and HU group were administered saline alone. The allopurinol group was administered allopurinol (5 mg/kg B.W., Sigma-Aldrich, Saint Louis, MO, USA). The body weight was recorded every 3 days.

Mice were monitored daily, and parameters such as body weight, activity, appearance, and posture were recorded twice a week to evaluate the comfort of animals and give them scores from 1 to 5. Any animal that scored higher than 3 (weight loss higher than 20% compared to other animals in the same cage, hair problems, or abnormal activity) were separated and immediately euthanized. After 21 days of treatment, mice were euthanized after anesthesia induction with 3% isoflurane by cervical dislocation and organs were sectioned for analysis.

#### 2.4.2. Serum Biochemical Analysis

Blood samples were collected from the heart on day 21. The collected samples were immediately moved to ice and after 1 h centrifuged at 2000× *g* for 15 min at 4 °C. The serum was separated and stored at −80 °C until analyses. The levels of uric acid, creatinine, and blood urea nitrogen (BUN) were measured using a biochemical blood analyzer ((FUJIFILM DRI-CHEM NX500i, Tokyo, Japan). The xanthine oxidase levels in the serum of each group were measured using a xanthine oxidase activity assay kit (Sigma-Aldrich, Saint Louis, MO, USA). 

#### 2.4.3. Kidney, Intestine Sectioning and Histopathological Assessment

After euthanasia, the intestine and right kidney from each animal was immediately removed, rinsed, and fixed in 4% paraformaldehyde (Sigma-Aldrich, Saint Louis, MO, USA) for 24 h, followed by paraffin embedding; each paraffin block was sectioned (5 µm) for Hematoxylin and Eosin (H & E), and alcian blue staining. Paraffin-embedded kidney samples were sectioned and stained with H & E for pathological evaluation, glomerular congestion, matrix expansion, and inflammation. Paraffin-embedded intestine samples were sectioned and stained with alcian blue for evaluation of acidic epithelial and connective tissue mucins.

For quantification of glomerulus morphology changes and hypertrophy, the glomerular image was digitally sectioned into 20 images and in each of them, two glomeruli, in which the vascular pole was evident, H & E samples were sectioned by 25 different slots in a virtual grid and two glomeruli from each image were selected by the previous criteria for mesangial area measurement. If some slots did not include any glomeruli, additional glomeruli from the next slot were added to give the same number of glomeruli per sample. The areas were selected and assessed using the “draw closed polygon” tool in CaseViewer by 3DHISTECH (Budapest, Hungary). All areas were recorded and analyzed.

#### 2.4.4. Fecal Sample Analysis

After sacrifice, the intestine was immediately dissected, and the feces were collected in a tube and stored at −80 °C before use. For metagenome analysis, DNA was extracted with the Exgene^TM^ Stool DNA mini kit (GeneAll, Seoul, Korea) according to the manufacturer’s protocol. The V3-V4 region of the bacterial 16S rRNA gene was amplified by using barcoded universal primers 341F and 805R [33]. Microbiome profiling was conducted with the 16S-based Microbial Taxonomic Profiling platform of EzBioCloud Apps (Sanigen, Anyang, Korea).

For extraction of short-chain fatty acids in the fecal sample, 0.5 g of each of the fecal samples was thawed, suspended in 2 mL of DW, and homogenized for 5 min. The pH of the suspension was adjusted to 3 by adding 5 M HCl and kept at room temperature for 10 min with occasional shaking. The suspension was then centrifuged at 5000 rpm for 20 min, and the supernatant was used for gas chromatography-mass spectrometry (GC-MS) analysis (Thermo Fisher Scientific, Dreieich, Germany). The content of short chain fatty acids (SCFAs) in each sample was then calculated by comparing them with the standard.

#### 2.4.5. RNA Extraction, cDNA Synthesis, and Quantitative Real-Time PCR (qRT-PCR)

Total RNA was isolated with an RNA isolation kit (Takara, Dalian, China) according to the manufacturer’s instructions. Agarose gel electrophoresis of RNA samples confirmed its integrity. RNA concentration and purity were determined by an ND-1000 spectrophotometer. Before cDNA synthesis, gDNA was removed from the RNA preparation by using a Takara PrimeScript RT reagent kit with a gDNA eraser (Takara, Dalian, China). cDNA was synthesized according to the manufacturer’s instructions. cDNAs were mixed with 10 μL of SYBR Premix Ex Taq (Takara, Dalian, China) and 0.5 µM of each primer pair in a 20 μL final volume and subjected to 45 PCR cycles (95 °C for 3 s and 60 °C for the 20 s) with the Roche LightCycler^®^ 96 System. GAPDH, a housekeeping gene, was used as a reference to normalize expression levels and to quantify changes in uric acid-associated gene expression between non-treated and treated groups. The relative mRNA levels of genes of interest were determined and normalized to the expression of the housekeeping gene using 2^−ΔΔCT^ value analysis. The sequences of primers for qRT-PCR are shown in Table 1.

#### 2.4.6. ZO-1 and Occludin Expression

The intestine tissue samples were collected on day 21. The collected samples were immediately moved to liquid nitrogen and stored at −80 °C until analyses. The amount of ZO-1 expression was measured using a Mouse TJP1/ZO-1 enzyme-linked immunosorbent assay (ELISA) kit (LSBio, Seattle, WA, USA). The amount of occludin expression was measured using a Occludin ELISA kit (Antibodies-Online Inc., Pottstown, PA, USA).

### 2.5. Statistical Analysis

All data are shown as mean ± SD. The statistical difference between the control and experimental groups was analyzed with a one-way analysis of variance (ANOVA), or two-sample *t*-test to determine statistical significance. All statistical analyses were carried out with GraphPad Prism software (version 8.0). *p* values between groups of 0.05 or less were considered statistically significant. All experiments were repeated 3 times.

## 3. Result

### 3.1. Screening of Inosine, Guanosine, and Adenosine Degrading LAB Strains

#### 3.1.1. Assimilation of Guanine and Inosine by LAB Strains

To screen potential probiotic strains, with anti-hyperuricemia activity, a total of 22 LAB strains isolated from fermented food were examined for inosine and guanosine degrading abilities by HPLC. A decomposition rate of guanine and inosine close to 100% could be observed in the MJM60396 and MJM60662 strains (Table 2).

#### 3.1.2. Degradation of Purine Compounds by Cell Lysates

To explore the purine-degrading effect of cell lysates, strain MJM60396 and MJM60662 were lysed and centrifuged. The supernatant contained the cytoplasmic fraction, and the pellet contained the membrane debris. The effects of live cells, cell lysis pellets, and cell lysis supernatants on the decomposition of nucleosides are shown in Table 3. The live cells of MJM60396 and MJM60662 strains completely degraded all purine nucleotides. The lysis pellet of MJM60396 degraded 100% of adenosine, guanosine, and inosine. The lysis supernatant of MJM60396 degraded 29% of adenosine, 48% in guanosine, and 50% of inosine.

### 3.2. Phylogenetic Analysis of MJM60396

**Figure 1:**
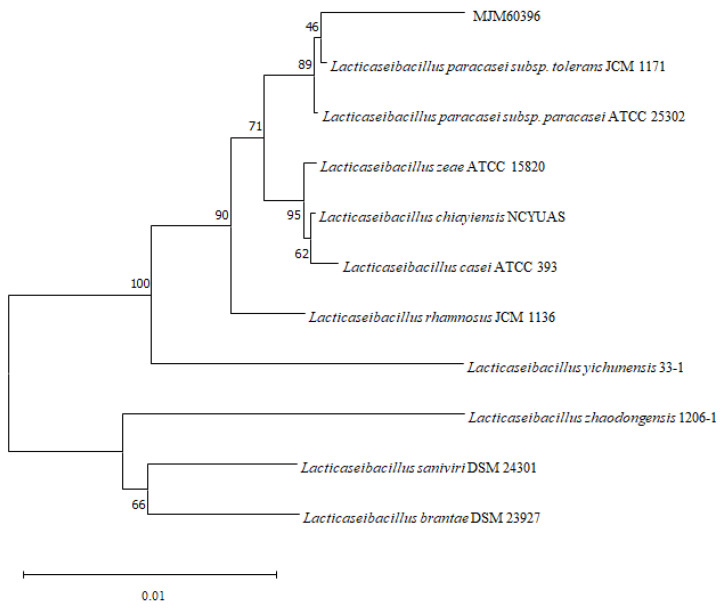
Phylogenetic analysis of MJM60396. Phylogenetic analysis was performed by the neighbor-joining method, using MEGA11 software. Numbers in the branches represent the bootstrap values (%) from 1000 replicates. The evolutionary distances were computed using the *Kimura* 2-parameter method. The scale bar indicates 0.01 substitutions per nucleotide position.

In order to accurately identify the species of the bacteria isolated from fermented food, the 16rDNA gene was sequenced and phylogenetic analysis was performed. A blast search of the Ezbiocloud database showed that the 16S rDNA sequence of the isolates were identical to *Latilactobacillus, Leuconostoc, Lacticaseibacillus, Levilactobacillus* species. MJM60396, which showed the highest activity for degradation of purine compounds, showed 99% similarity with *L. paracasei* subsp. *tolerans* JCM 1171 (Table 2). Phylogenetic analysis of the 16S rRNA gene sequence of MJM60396 with that of various *Lactobacillus* species confirmed that it is closely related to *L.* *paracasei* (Figure 1).

### 3.3. Utilization of Purine Compounds by LAB for Growth

**Figure 2:**
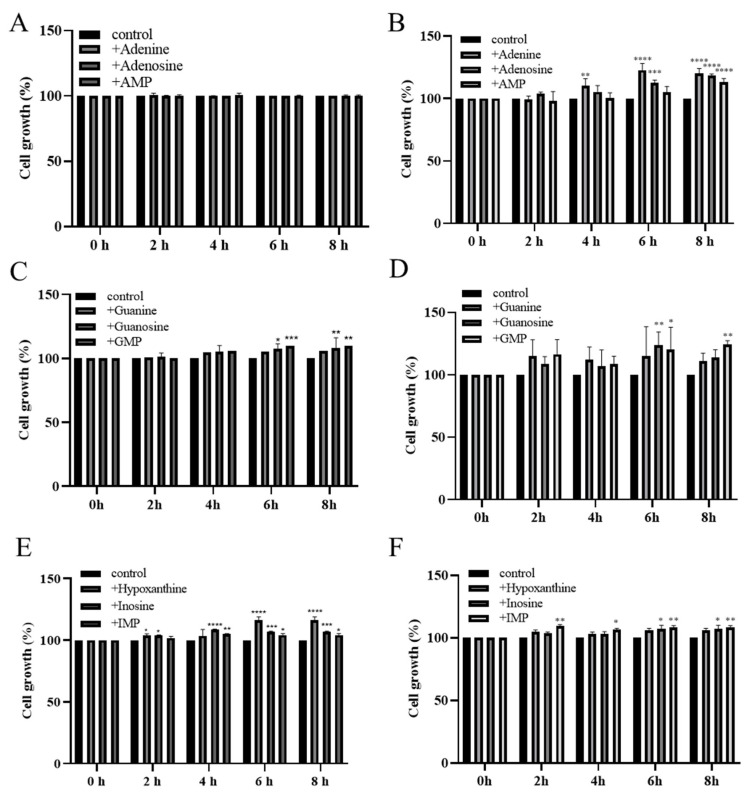
The growths of LAB increased in the medium supplemented with purine nucleotides, nucleosides, and bases. MJM60396 was incubated in the medium supplements with AMP, adenosine, and adenine (**A**), GMP, guanosine and guanine (**C**), IMP, inosine, and hypoxanthine (**E**). MJM60662 was cultured in medium supplements with AMP, adenosine, adenine (**B**), GMP, guanosine and guanine (**D**), IMP, inosine, and hypoxanthine (**F**). Values represent the means ± SD of three samples. * *p* < 0.05, ** *p* < 0.01, *** *p* < 0.001, **** *p* < 0.0001 compared with control.

To confirm the absorption and utilization of purine compounds by LAB, the growth of MJM60396 in a medium containing purine compounds was compared to the growth in the absence of purine compounds. Both MJM60396 and MJM60662 (positive control) grew in the DM medium supplemented with or without the purine compounds, but the growth was different. After being incubated for 8 h, the growth of MJM60396 increased by 0%, 0.2%, 0.3%, 5.7%, 8%, 9.9%, 16.4%, 6.8%, and 4.2% in the medium supplemented with adenine, adenosine, adenosine monophosphate (AMP), guanine, guanosine, guanosine monophosphate (GMP), hypoxanthine, inosine, and inosine monophosphate (IMP), respectively, compared with non-supplemented control (Figure 2A,C,E). The growth of MJM60662 increased in these media by 20%, 18.4%, 13.2%, 10.8%, 13.8%, 24.2%, 6.1%, 7%, and 8.2%, respectively (Figure 2B,D,F). Among these purine compounds, MJM60396 grew significantly in the medium supplemented with hypoxanthine (16.4%), while MJM60662 mostly utilized GMP (24.2%).

### 3.4. Animal Study

#### 3.4.1. Effect of MJM60396 on the Body Weight and the Serum Uric Acid Level

**Figure 3:**
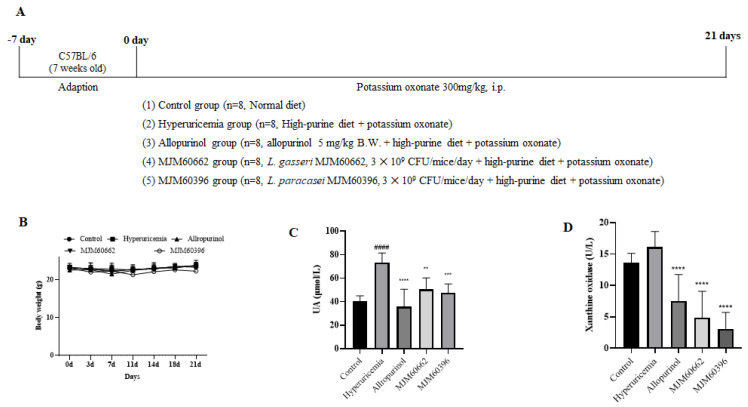
The animal study (**A**) Schematic representation of the experimental schedule. Control group, mice fed with the normal diet; Hyperuricemia group, mice fed with the high−purine diet + Oxonate 300 mg/kg; Allopurinol group, mice fed with the high−purine diet + Oxonate 300 mg/kg + allopurinol 10 mg/kg; MJM60662 group, mice fed with the high−purine diet + Oxonate 300 mg/kg + *L. gasseri* MJM60662 strain; MJM60396 group, mice fed with the high−purine diet + Oxonate 300 mg/kg + *L. paracasei* MJM60396. The results are presented as mean ± SD. (**B**) The body weights of mice. (**C**) The uric acid level in serum. (**D**) Serum xanthine oxidase was analyzed by ELISA. #### *p* < 0.0001 compare with control group, ** *p* = 0.01, *** *p* < 0.001, **** *p* < 0.0001 compare with hyperuricemia group.

An animal study was performed to explore the anti-hyperuricemia activity of MJM60396 in mice. For the animal study, hyperuricemia was induced by intraperitoneal injection of potassium oxonate and a high-purine diet. MJM60396 and MJM60662 were supplemented with hyperuricemia mice for 21 days (Figure 3A). The control group was fed a normal diet, and the other groups were fed a high-purine diet to increase the purine uptake from food. The hyperuricemia group served as the symptom control, and the allopurinol group and MJM60662 group were used as positive controls. Bodyweight was checked every 3 days.

Blood samples were taken on day 21. As shown in Figure 3B, body weight gain was not significantly different among all groups. The uric acid level of the hyperuricemia group increased significantly at 73.36 μmol/L. The uric acid in hyperuricemia increased almost two-fold compared with control (40.64 μmol/L), indicating the successful establishment of hyperuricemia in mice. The serum uric acid levels were 40.64, 73.36, 35.6, 50.56, and 47.58 μmol/L for the control, hyperuricemia group, allopurinol group, MJM60662 group, and MJM60396 group, respectively. Administration of MJM60396 significantly decreased the serum level by 35% compared with the hyperuricemia group (Figure 3C).

Xanthine oxidase is an enzyme that converts hypoxanthine into xanthine in uric acid. The amount of uric acid increases when there is a large amount of XO. The XO level was detected as 13.65 U/L in the control group, 16.08 U/L in the hyperuricemia group, 7.5 U/L in the allopurinol group, 4.88 U/L in the MJM60662 group, and 3.11 U/L in the MJM60396 group (Figure 3D). XO levels in the MJM60396 group were 81% lower than in the hyperuricemia group.

#### 3.4.2. Effect of LAB on the Kidney by Histological Observation and Biochemistry Analysis

**Figure 4:**
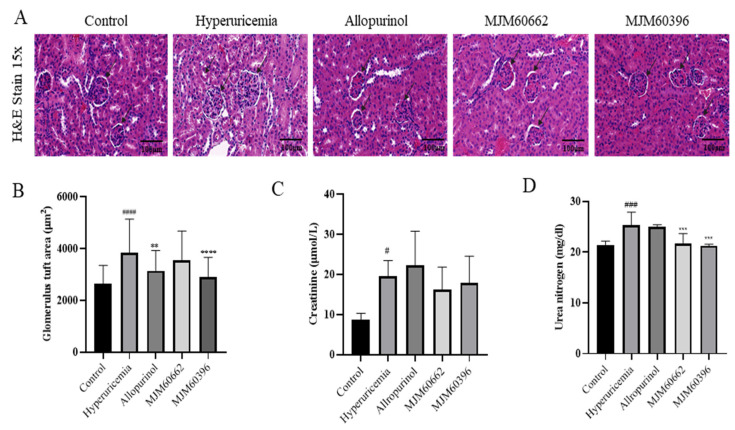
Kidney H&E staining and creatinine, urea nitrogen level in serum. (**A**) Effect of MJM60396, commercial strain and allopurinol on renal histopathology of hyperuricemia mice at magnification of 15× H & E staining. (**B**) The level of glomerulus tuft area. (**C**) The level of serum creatinine. (**D**) The level of serum urea nitrogen. Data are presented as means ± SE of 6 different mice. # *p* < 0.05, ### *p* < 0.001, #### *p* < 0.0001 compare with control group, ** *p* = 0.01, *** *p* < 0.001, **** *p* < 0.0001 compared with hyperuricemia group. Scale bar: magnification of 15× = 100 μm.

Around 70% of uric acid is excreted from the kidneys. For this reason, an increase in uric acid levels in serum can cause changes in kidney structures such as the glomerulus, Bowman’s capsule, and tubules [34]. Our study showed a decrease in the Bowman’s capsule space and dilatation of the proximal tubule’s cells. These morphological changes were in the hyperuricemia group but attenuated in the MJM60396 group, which showed an increase in the Bowman’s capsule space and the shape of each glomerulus tended to be more circumference styled (Figure 4A).

To avoid any subjective evaluation of the analysis of the kidney’s pathological alteration, we proceeded to calculate the glomerulus tuft area of each sample. This area measurement is related to the levels of hypertrophy shown for each glomerulus, in which a more hypertrophic structure will be larger compared with non-treated animals [35,36]. The hyperuricemia group showed a glomerular tuft area of 3842.07 µm^2^, representing an increase of 46% compared with the control group. The control group showed a glomerular tuft area of 2630.16 µm^2^. The allopurinol and MJM60662 groups showed a glomerular area of 3147.25 µm^2^ and 3551.22 µm^2^, respectively, which was a 19.6% and 35% increase, respectively, compared with the control group. Finally, MJM60396-treated mice showed 2901.86 µm^2^, which represents a significant decrease of the glomerular area by 24.5% when compared with the hyperuricemia group. This result confirms the effectiveness of MJM60396 to attenuate the morphological changes present in hyperuricemia-treated mice in the kidney and increase the percent of the correct functioning of the organ (Figure 4B).

Creatinine and blood urea nitrogen indicate kidney function and show malfunctions when their levels increase in serum. As shown in Figure 4C, creatinine was significantly increased in the hyperuricemia group compared with the control. In contrast, creatinine levels in the MJM60396 (17.82 μmol/L) and MJM60662 (16.2 μmol/L) group were lower compared with the hyperuricemia group (19.59 μmol/L). Blood urea nitrogen levels are shown in Figure 4D; the MJM60662 group was 21.73 mg/dl and the MJM60396 group was 21.73 mg/dL. Both results showed that levels were significantly lower in MJM60396 groups compared to the hyperuricemia group (25.36 mg/dL), indicating a higher function of the kidney compared to the hyperuricemia group.

#### 3.4.3. Effect of MJM60396 on mRNA, Protein Expression of Kidney Genes Associated with Hyperuricemia

**Figure 5:**
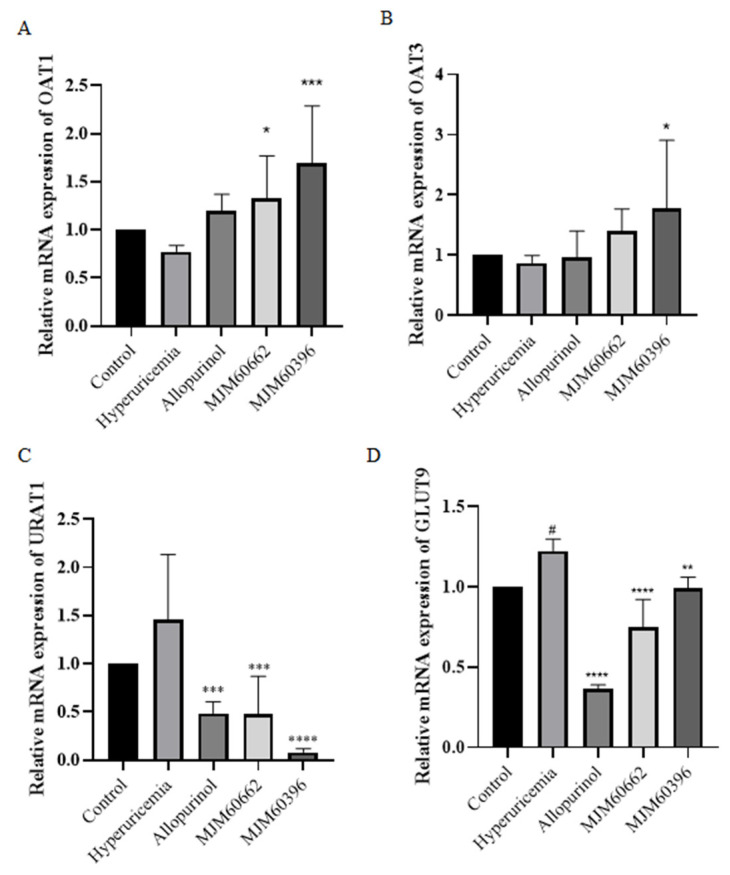
The effect of *L. paracasei* MJM60396 on mRNA and protein expression of hyperuricemia mice. (**A**) The mRNA expression of mOAT1 in mice kidneys was analyzed by RT-PCR. (**B**) The mRNA expression of mOAT3 in mice kidneys. (**C**) The mRNA expression of mURAT1 in mice kidneys (**D**) The mRNA expression of mGLUT9 in mice kidneys. # *p* < 0.05 compared with control group, * *p* < 0.05, ** *p* < 0.01, *** *p* < 0.001, **** *p* < 0.0001 compared with hyperuricemia group.

The uric acid transporters OAT1 and OAT3 play an important role in the transport of uric acid in the tubules [37]. It is known that hyperuricemia is caused by dysfunction of the urate transporters OAT1, OAT3, URAT1, and GLUT9 [38]. The gene expression levels of mOAT1 and mOAT3 were slightly lower in the hyperuricemia group compared to the control group (Figure 5A,B). Compared with the hyperuricemia group, the gene expression level of mOAT1 and mOAT3 notably increased in the MJM60396 group. The gene expression levels of URAT1 and GLUT9 increased in the hyperuricemia group compared with the control group (Figure 5C,D). Compared to the hyperuricemia group, the levels of URAT1 and GLUT9 significantly decreased in the allopurinol group, MJM60662, and MJM60396 groups.

#### 3.4.4. Alcian Blue Staining and Expression of Tight Junction Proteins in the Intestine

**Figure 6:**
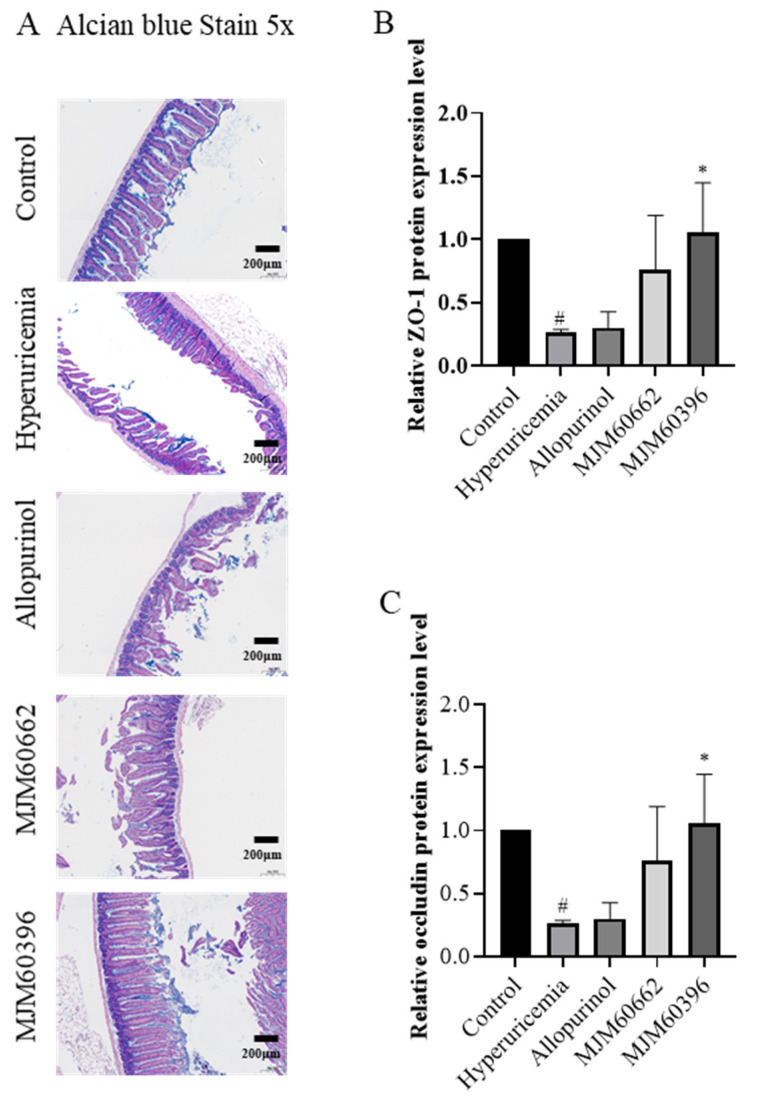
Intestine alcian blue staining and expression of tight junction proteins in the intestine. (**A**) Effect of MJM60396, MJM60662 strain, and allopurinol on intestine histopathology of hyperuricemia mice at a magnification of 5× alcian blue staining. (**B**) Protein expression levels of intestine ZO-1 were analyzed by ELISA. (**C**) Protein expression levels of intestine occludin were analyzed by ELISA. # *p* < 0.05 compare with control group. * *p* < 0.05 compared with hyperuricemia group. Scale bar: magnification of 5× = 200 μm.

Damaged intestinal barrier and increased intestinal permeability are characteristic of hyperuricemia mice. Alcian blue staining was conducted to analyze the impaired intestinal barrier. Our results showed that the intestines of the hyperuricemia and allopurinol group exhibited defects including sparse intestinal villi, mucosal and submucosal edema when compared to the control group (Figure 6A). In contrast, the MJM60396 group showed normal intestinal structure, villi, and no submucosal edema, which was similar to the control group.

The protein expression levels of ZO-1 in the intestinal tissues were downregulated in the hyperuricemia, allopurinol, and MJM60662 groups compared with the control as determined by ELISA. The intestinal ZO-1 level had recovered in the MJM60396 group (Hyperuricemia vs. MJM60396, *p* = 0.0154) (Figure 6B). The expression of occludin in the hyperuricemia group significantly decreased compared with the control group (Control vs. Hyperuricemia, *p* = 0.0232). Although the expression of occludin was not recovered in the allopurinol and MJM60662 groups, it was recovered in the MJM60396 groups compared with the hyperuricemia group (Hyperuricemia vs. MJM60396, *p* = 0.0154) (Figure 6C).

#### 3.4.5. Intestinal Microbial Diversity

**Figure 7:**
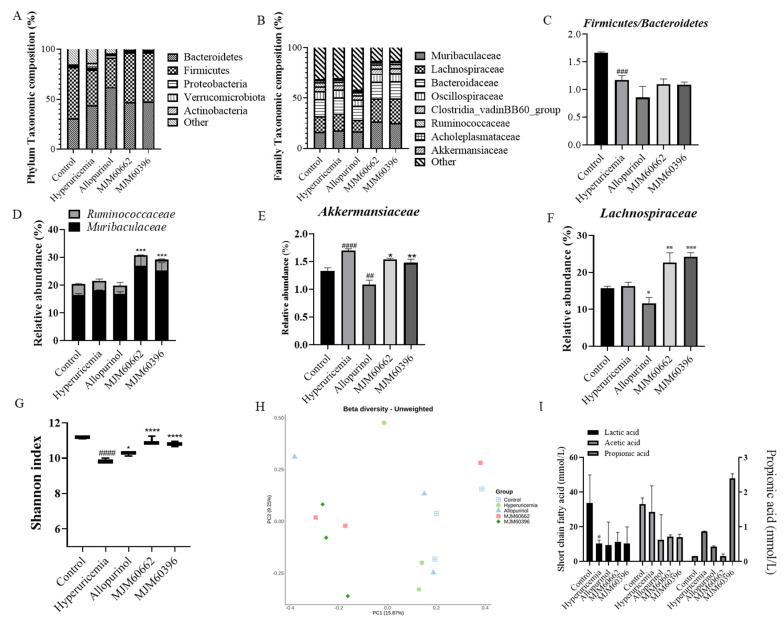
LAB supplementation modulated the composition of the gut microbiota. (**A**) Taxonomic composition at the phylum level. (**B**) Taxonomic composition at the family level. (**C**) *Firmicutes/Bacteroidetes* (F/B) ratio (**D**) Relative abundance of *Muribaculaceae* and *Ruminococcaceae*. (**E**) Relative abundance of *Akkermansiaceae*. (**F**) Relative abundance of *Lachnospiraceae*. (**G**) Alpha−diversity is indicated by the Shannon index. (**H**) Beta-diversity comparisons. The colors of the boxplots and dots represent the different groups analyzed according to the legend. (**I**) The amount of SCFAs was measured in the feces of each group. Quantities of lactic acid, acetic acid, propionic acid, were measured by GC−MS. ## *p* < 0.01 ### *p* < 0.001, #### *p* < 0.0001 compared with control group, * *p* < 0.05, ** *p* < 0.01, *** *p* < 0.001, **** *p* < 0.0001 compared with the hyperuricemia group.

The association between LAB intake and changes in the gut microbiota was evaluated at the phylum and family level. At the phylum level, *Bacteroidetes, Firmicutes, Proteobacteria, Verrucomicrobiota*, and *Actinobacteria* constituted the main microbiota, of which *Bacteroidetes* and *Firmicutes* were dominant (Figure 7A). The portions of *Bacteroidetes, Firmicutes, Proteobacteria, Verrucomicrobiota*, and *Actinobacteria* were 30.88, 51.44, 1.02, 1.33 and 0.032%, respectively, in the control group, and 44.15, 35.32, 1.75, 1.70, and 3.45% in the hyperuricemia group; 62.07, 29.66, 2.46, 1.09 and 0.46% in the allopurinol group, 47.11, 49.42, 1.05, 1.54, and 0.04% in the MJM60662 group, and 47.42, 49.42, 0.91, 1.48, and 0.13% in the MJM60396 group. In the hyperuricemia group, *Proteobacteria, Verrucomicrobiota*, and *Actinobacteria* increased slightly compared to the control group.

At the family level, the mice microbiota was mainly composed of *Muribaculaceae*, *Lachnospiraceae*, *Bacteroidaceae*, *Oscillospiraceae*, Clostridia_vadinBB60_group, *Ruminococcaceae*, *Acholeplasmataceae*, and *Akkermansiaceae* (Figure 7B). The relative portions for *Muribaculaceae*, *Lachnospiraceae*, *Bacteroidaceae*, *Oscillospiraceae*, Clostridia_vadinBB60_group, *Ruminococcaceae*, *Acholeplasmataceae*, and *Akkermansiaceae* were 16.49, 15.73, 16.93, 7.62, 4.92, 3.93, 2.10 and 1.33% in the control groups, 18.14, 16.27, 16.21, 7.64, 4.67, 3.51, 2.00 and 1.70% in the hyperuricemia group, 16.84, 11.61, 13.79, 6.51, 4.19, 3.01, 1.70 and 1.09% in the allopurinol group, 26.93, 22.62, 16.53, 8.26, 5.13, 3.83, 2.08 and 1.54% in the MJM60662 group, and 25.43, 24.19, 17.16, 8.03, 5.22, 3.78, 2.04 and 1.48% in the MJM60396 group, respectively. The MJM60396 group showed an increase in all flora compared to the hyperuricemia group except for *Akkermansiaceae*.

The *Firmicutes/Bacteroidetes* ratio significantly decreased in the hyperuricemia group compared to the control group; the ratio was not reversed in the treatment groups, but was low when allopurinol was taken (Figure 7C). At the family level, the relative abundance of *Ruminococcaceae* and *Muribaculaceae* in MJM60662 and MJM60396 groups significantly increased compared to the hyperuricemia group (Figure 7D). The relative abundance of *Akkermansiaceae* in the hyperuricemia group significantly increased compared to the control group, while the MJM60662 and MJM60396 groups showed significantly decreased abundance compared to the hyperuricemia group (Figure 7E). The relative abundance of *Lachnospiraceae* significantly increased in the MJM60662 and MJM60396 groups compared to the hyperuricemia group (Figure 7F).

The Shannon Diversity Index was evaluated to determine the gut microbial alpha diversity (Figure 7G). The Shannon index was significantly lower in the hyperuricemia group than in the control group. There was a slight increase in the allopurinol group compared to the hyperuricemia group, and the Shannon index significantly increased for the groups taking MJM60662 and MJM60396. To analyze beta diversity, the distance for each sample was calculated using the presence or absence of microbial flora (unweighted) and displayed in the coordinate space. As a result of beta diversity analysis, it was confirmed that the intake of *L. paracasei* MJM60396 can affect the composition of the microbiota (Figure 7H). The MJM60396 group showed a significant change in the composition of the intestinal microbiota compared to the control group. The gut microbiota between mice fed *L. paracasei* MJM60396 and those without it was markedly different.

The amount of SCFAs in the feces was measured in each group (Figure 7I). The lactic acid, acetic acid, and propionic acid were quantified by GC-MS. The lactic acid content was 33.04 mmol/L in the control group, 10.35 mmol/L in the hyperuricemia group, 9.47 mmol/L in the allopurinol group, 11.28 mmol/L in the MJM60662 group, and 10.18 mmol/L in the MJM60396 group. The acetic acid content was 33.69 mmol/L in the control group, 28.46 mmol/L in the hyperuricemia group (*p* = 0.0417), 12.41 mmol/L in the allopurinol group, 14.27 mmol/L in the MJM60662 group, and 14.01 mmol/L in the MJM60396 group. The content of propionic acid was 0.15 mmol/L in the control group, 0.87 mmol/L in the hyperuricemia group, 0.37 mmol/L in the allopurinol group, 0.14 mmol/L in the MJM60662 group, and 2.38 mmol/L in the MJM60396 group.

## 4. Discussion

Purine-rich foods, meat, seafood, and alcohol can aggravate hyperuricemia [1]. However, it is not recommended for asymptomatic hyperuricemia patients to take drugs to reduce serum uric acid levels. If these patients are not able to be treated on time, they are more likely to undergo urgent surgery or hospitalization in the future. Gout occurs when the serum UA level is 7.0 mg/dL or higher, and in those with a strong family history and who are relatively young. It is necessary to find a safe way to manage hyperuricemia to prevent gout. Taking probiotics is recognized as an alternative way to prevent hyperuricemia and gout. Among the probiotic strains, *Lactobacillus* is the main bacteria that showed diverse biological functions. Some *Lactobacillus* were reported to show anti-hyperuricemia activity in recent studies. A lactic acid bacteria, *Lactobacillus brevis* DM9216 isolated from Chinese sauerkraut, can degrade purine compounds and lower the uric acid level in the serum in an animal study [39]. *Lactobacillus gasseri* PA3 can utilize the purine compounds for growth, and decrease the purine absorption in rats [15].

In the present study, we screened 22 *Lactobacillus* strains for their ability to degrade guanosine and inosine in vitro. HPLC analysis showed that the assimilation rates of guanosine and inosine were shown by most of the strains evaluated. However, the assimilation rate was different. *L. mesenteroides* MJM60367 showed a great guanosine assimilation rate of 92.6% but exhibited a very low inosine assimilation rate of 25.1%. Even for the same species, the assimilation rates were different. *L. paracasei* MJM60396 can almost completely assimilate both guanosine and inosine, but *L. paracasei* MJM60341 showed only a moderate assimilation rate of guanosine (73.3%) and inosine (44.3%). These results show that the purine degradation efficiency is strain-specific rather than species-specific. Therefore, MJM60396 was selected for its superior purine assimilation rate. *Lactobacillus gasseri* PA3 (named MJM60662 in this study), isolated from a commercial yogurt, which showed anti-hyperuricemia activity, served as a control in this study [40,41].

One mechanism by which *Lactobacillus* degrades purines is that LAB use purines for growth, reducing their absorption in the host. *L. gasseri* PA-3 is effective in preventing hyperuricemia by consuming IMP, inosine, and hypoxanthine, and lowering uric acid levels in the serum [15]. Another study reported that the uptake of nucleotide precursors stimulated the growth of *L. lactis* [16,42]. Therefore, comparing the growth rate of *Lactobacillus* in the presence and absence of purine compounds can demonstrate the utilization of purines for growth by lactic acid bacteria. In a utilization assay by using various purine compounds, both MJM60396 and MJM60662 showed a higher growth rate in the presence than in the absence of the purine compounds. The difference is that MJM60396 prefers hypoxanthine, and MJM60662 prefers adenine. These results confirmed the utilization of the purine compound by *L. paracasei* MJM60396. Maintaining a low concentration of purine compounds is helpful to decrease serum uric acid. One of the mechanisms by which allopurinol reduces uric acid is to decrease the phosphoribosylpyrophosphate concentration, thereby reducing purine synthesis to lower purine concentration [43]. This is similar to the way that MJM60396 uses purines for growth and reduces purine absorption by the host from food, thus lowering the concentration of purines in the body. MJM60396 is effective in consuming various purine compounds, including IMP, inosine, hypoxanthine, AMP, adenosine, adenine, GMP, guanosine, and guanine. In particular, the MJM60396 grew 16% higher in the presence of hypoxanthine than in the absence of hypoxanthine. The results of the utilization assay confirmed the purine absorption and utilization by MJM60396, which may contribute to the uric acid lowering effect.

Further experiments were carried out to determine whether the cell lysates (cell lysates supernatant, and cell lysates pellet) had a purine-degrading effect, and the effects were compared with that of a live cell. The live cells showed the best purine-degrading activity. The lysates’ supernatant and pellet also showed degrading activity, the degrading activity for the lysates’ pellet was higher than that of the lysates’ supernatant. The components in the cell membrane and cytoplasm, such as organelles and crude enzymes, may be related to the degrading activity [44]. The development of the probiotic product by using cell lysates has a great advantage in decreasing product cost.

To be developed as a probiotics product for lactic acid bacteria, it is very important to assess its safety and probiotics characteristics (Tables S1 and S2). *Lactobacillus* has a DL-lactate racemase to convert L-lactate to D-lactate, which cannot be metabolized. The accumulation of D-lactate may cause acidosis in babies, children, or short bowel syndrome patients [45]. Some LAB can deconjugate bile salt to protect themselves to survive in the intestinal environment. However, too much bile salt deconjugation may inhibit fat emulsification, and the deconjugated bile salt may act on some other intestinal microorganisms to produce toxic secondary metabolites. Some *Lactobacillus* can produce biogenic amines, which can have toxic effects on humans [46]. We observed that the MJM60396 strain did not produce any D-lactate or biogenic amine, and the bile salt deconjugation was negative (Table S1 in Supplementary Materials). In addition, MJM60396 showed no hemolytic activity. In the antibiotic susceptibility test, MJM60396 was sensitive to most of the antibiotics except for kanamycin. Resistance of *Lactobacillus* to aminoglycoside antibiotics is considered unique and non-transmissible. Survival of *Lactobacillus* strains in the digestive system and colonization of the intestinal epithelium is essential for probiotic activity. In the oral-gastrointestinal transit assay, MJM60396 maintained high viability. HT-29 cells are used to study the intestinal epithelial adherence of probiotic strains. MJM60396 showed a cell adhesion rate of 4.4%. These results conferred MJM60396 with probiotic characteristics, and it can be used as a probiotic supplement. Furthermore, MJM60396 showed strong antibacterial activity against many enteric pathogens (Table S2). These enteric pathogens induce intestinal toxins and cause diarrhea, and destroy the intestinal barrier by causing loss of tight junction proteins such as ZO-1 and occludin [47]. MJM60396 showed an antibacterial effect on enteric pathogens, thus protecting the intestinal barrier.

The anti-hyperuricemia activity of MJM60396 was further investigated in a mouse model. Hyperuricemia was induced by supplementation with excessive purine compounds and blockage of uricase through high purine diets and i.p. injection of potassium oxonate. The uric acid level increased two-fold in the hyperuricemia group, and the administration of MJM60396 resulted in a significant decrease in the serum uric acid level. The uric acid level can be determined by the synthesis and excretion of uric acid, as well as the purine absorption.

Xanthine oxidase is the key enzyme for the synthesis of uric acid; it can oxidize hypoxanthine and xanthine, releasing uric acid [48]. Therefore, reducing the amount of XO can also help reduce hyperuricemia and gout. Although MJM60396 was selected for its high purine degrading activity, mice fed with MJM60396 showed 81% reduced activity of XO in serum compared with the hyperuricemia group. The mechanism of LAB regulating XO is not well understood. Recently, Ni et al. reported that oral administration of LAB to mice suppressed serum and hepatic XO [49]. The authors suggested two mechanisms for the suppression of XO by oral administration of LAB in hyperuricemia mice. One was the regulation of intestinal microbiota to promote the production of short-chain fatty acids, which can suppress XO directly. Another was that SCFAs indirectly suppress XO by maintaining the gut barrier function to prevent the translocation of toxic compounds, like lipopolysaccharide (LPS), to the liver and blood, as both LPS and pro-inflammatory cytokines have been reported to increase hepatic XO activity.

In our study, the microbiota composition was changed in the treatment groups. Analysis of the taxonomic composition showed that *Muribaculaceae* and *Lachnospiraceae* were enriched in the MJM60396 group. *Muribaculaceae* and *Lachnospiraceae* are commensals that compete with pathogens for mucus-derived sugars, therefore ecologically maintaining a healthy gut [50]. Although *Muribaculaceae* and *Lachnospiraceae* are also involved in the metabolism of short-chain fatty acids such as propionate [51,52], the main role of these bacteria in this study may be as a competitor of pathogens for nutrients because the SCFAs did not change significantly. The *Akkermansiaceae* family of the *Verrucomicrobia* phylum is a mucin-degrading microorganism [53]. It was reported that a decrease in *Akkermansia muciniphila* is associated with an increased risk of inflammatory bowel diseases [54]. While another study reported that an increase in *Akkermansiaceae* might be associated with Crohn’s disease [55,56]. It seems very important to control *Akkermansiaceae* at a suitable level to maintain the integrity of the intestinal barrier. In this study, the hyperuricemia group showed a significantly higher abundance of *Akkermansiaceae* than the control group. On the other hand, the group taking allopurinol had significantly lower *Akkermansiaceae* abundance than the control group. The dysbiosis of *Akkermansiaceae* abundance in the hyperuricemia and allopurinol group might be associated with the impaired intestinal barrier and the low expression levels of tight junction proteins, while the supplementation of LAB alleviates the dysbiosis to a certain extent. Moreover, MJM60396 increased the intestinal microbial alpha diversity, and the beta diversity showed a distinct difference between the treatment groups and the hyperuricemia groups. Hyperuricemia was reported to be associated with impaired intestinal permeability in mice [29]. The breakdown of tight junction protein contributed to an increase in intestinal permeability in response to the stimulation of inflammatory cytokines [57]. The enhancement of tight junction protein expression by *Lactobacillus* spp. was related to the activation of the Toll-like receptor 2 signaling pathway, and the activation is strain-specific [58]. According to our results, supplementation of MJM60396 significantly increased the expression of intestinal ZO-1 and occludin, whose expression was reduced in the hyperuricemia group. This result demonstrated that MJM60396 increased the integrity of the intestinal barrier by enhancing the expression of tight junction proteins. All these data suggested that supplementation of LAB improved gut health, which might contribute to the relief of inflammation-associated hyperuricemia.

Another mechanism for lowing serum uric acid is the enhancement of urate excretion. The kidney is the main organ responsible for excreting uric acid. It is excreted in the glomerular filtration, and reabsorbed in the proximal tubule by different urate transporters. The urate reabsorption transporters include URAT1, OAT4, OAT10, and GLUT9, and the urate excretion transporters include OAT1, OAT3, MRP2, MRP4, NPT1, and UAT. Hyperuricemia may be caused by decreased excretion/secretion of urate [38,59]. OAT1 and OAT3 are important for the consistent movement of uremic solutes across tissues and into the kidney [60]. In a previous study, it was shown that the expression levels of OAT1 and OAT3 were low in mice induced with hyperuricemia [61]. In this study, MJM60396-treated mice significantly increased the expression of mOAT1 and mOAT3. URAT1 and GLUT9 are located at the membrane of renal tubular epithelial cells responsible for uric acid reabsorption. In our results, the expression of mURAT1 and mGLUT9 was lowered in MJM60396-treated mice compared with the hyperuricemia group, indicating the decreased reabsorption of urate. Our results showed that MJM60396 can also modulate serum uric acid levels by enhancing uric acid excretion and inhibiting uric acid reabsorption in the renal tubule.

In addition, hyperuricemia can lead to the destruction of renal structure and function. It was reported that hyperuricemia mice developed glomerular hypertrophy, which leads to compression of the renal capillaries described as glomerular endothelins [62]. The histopathological results in our study showed that administration of MJM60396 decreased the glomerular tuft area compared with the hyperuricemia group. This confirmed that MJM60396 could attenuate the renal damage caused by hyperuricemia and improve renal function. The hyperuricemia mice showed increased creatinine and urea nitrogen level in serum, which are indicators of kidney toxicity. Treatment with MJM60396 decreased the creatinine and urea nitrogen level, suggesting that MJM60396 is a potential probiotic candidate that can safely lower uric acid levels and prevent hyperuricemia without causing kidney toxicity.

## 5. Conclusions

In this study, we isolated *L. paracasei* MJM60396 from fermented food as a probiotic candidate for the management of hyperuricemia due to its probiotic characteristics and anti-hyperuricemia activity in vitro and in vivo. MJM60396 decreased serum uric acid in multiple ways: (1) degrade and utilize purine compounds to prevent the excessive absorption of purine compounds from food by the host; (2) reduce the concentration of xanthine oxidase to decrease the synthesis of uric acid; (3) improve the kidney function and increase the uric acid excretion by regulating urate transporters; (4) recover the impaired intestinal barrier to reduce the permeability by enhancing the expression of tight junction proteins; (5) enhance the microbiota diversity to maintain healthy gut to reduce inflammation, which may aggravate hyperuricemia. In conclusion, these data suggested that MJM60396 can ameliorate hyperuricemia and thus can be a potential probiotic candidate for mediating hyperuricemia.

## Figures and Tables

**Table 1:** Primer sequences for qRT-PCR.

Gene	Primer	Sequence (5′-3′)
OAT1	Forward	GAGCAGAGGAAAGCAGAAGC
	Reverse	CCCTTTAGTGCTGTGTGACG
OAT3	Forward	TACAGTTGTCCGTGTCTGCT
	Reverse	CTTCCTCCTTCTTGCCGTTG
URAT1	Forward	AGGTCCTGACAGGTTCTGT
	Reverse	CTCTGCCTTCCTCCTGTTGA
GLUT9	Forward	TTCGGGTCCTCCTTCCTCTA
	Reverse	GGACACAGTCACAGACCAGA
GAPDH	Forward	GGCACAGTCAAGGCTGGAATG
	Reverse	ATGGTGGTGAAGACGCCAGTA

OAT1: organic anion transporter 1; OAT3: organic anion transporter 3; URAT1: solute carrier family 22; GLUT9: solute carrier family 2; and GAPDH: glyceraldehyde 3-phosphate dehydrogenase.

**Table 2:** Assimilation of nucleoside by candidate strains.

Strain No.	Strain Name *	$V_{gua}$ (μmol/min)	$D_{gua}$ (%)	$V_{\mathrm{ino}}$ (μmol/min)	$D_{ino}$ (%)
MJM60349	*Latilactobacillus curvatus*	1.7	16.6	1.5	14.3
MJM60355	*Latilactobacillus curvatus*	1.7	16.5	1.6	14.7
MJM60363	*Leuconostoc mesenteroides*	4.0	38.2	3.3	31.3
MJM60364	*Latilactobacillus sakei*	4.3	41.3	5.1	48.8
MJM60366	*Latilactobacillus curvatus*	10.3	100.0	2.9	27.9
MJM60367	*Leuconostoc mesenteroides*	9.6	92.6	2.7	25.1
MJM60368	*Latilactobacillus sakei*	3.4	33.1	3.4	32.5
MJM60370	*Leuconostoc mesenteroides*	2.7	25.5	2.3	22.2
MJM60371	*Leuconostoc mesenteroides*	4.8	46.1	4.0	38.0
MJM60375	*Latilactobacillus sakei*	4.0	39.0	4.2	40.2
MJM60376	*Leuconostoc mesenteroides*	1.9	18.4	1.9	18.1
MJM60377	*Leuconostoc mesenteroides*	1.9	18.6	1.9	17.7
MJM60378	*Leuconostoc mesenteroides*	6.5	62.4	5.3	50.4
MJM60341	*Lacticaseibacillus paracasei*	7.9	73.3	4.7	44.3
MJM60385	*Lacticaseibacillus rhamnosus*	2.8	30.0	2.1	23.0
MJM60386	*Levilactobacillus brevis*	6.9	75.9	6.3	67.5
MJM60389	*Lactobacillus plantarum*	7.5	82.1	6.8	73.0
MJM60391	*Levilactobacillus brevis*	4.1	44.8	3.0	32.5
MJM60392	*Lactococcus lactis*	1.9	21.0	2.0	21.1
MJM60396	*Lacticaseibacillus paracasei*	10.3	100.0	10.5	100.0
MJM60662	*Lactobacillus gasseri*	10.3	100.0	10.5	100.0
LGG	*Lacticaseibacillus rhamnosus* GG	8.5	81.16	8.2	78.53

*, Highest similarity based on the 16S rDNA sequence. Values are Means, *n* = 3. V_ino_, the incorporating speed of inosine by LAB strain; D_ino_, the incorporating rate of inosine by LAB strain; V_gua_, the incorporating speed of guanosine by LAB strain; D_gua_, the incorporating rate of guanosine by candidate strain.

**Table 3:** The degrading effect of live cells, cell lysis pellets, and cell lysis supernatants on adenosine, inosine, and guanosine.

Degradation (%)	*L. paracasei* MJM60396			*L. gasseri* MJM60662		
	Live	Pellet	Supernatant	Live	Pellet	Supernatant
Adenosine	100	100	29	100	45	44
Guanosine	100	100	48	100	72	46
Inosine	100	100	50	100	60	35

## Data Availability

The raw data used to support the findings of this study will be made available by the authors, without undue reservation, to any qualified researcher.

## Supplementary Materials

The following supporting information can be downloaded at: https://www.mdpi.com/article/10.3390/microorganisms10050851/s1, Table S1. In vitro probiotic characterization and safety assessment of *L. paracasei* MJM60396 and *L. gasseri* MJM60662; Table S2. Antibacterial activity of candidate LAB strains against enteric pathogens. References [63,64,65,66,67,68,69,70] cited in Supplementary Materials.
