# Supplementary material for: Potential Probiotic Lacticaseibacillus paracasei MJM60396 Prevents Hyperuricemia in a Multiple Way by Absorbing Purine, Suppressing Xanthine Oxidase and Regulating Urate Excretion in Mice
Source: Microorganisms. 2022 Apr 20;10(5):851. doi: 10.3390/microorganisms10050851 (PMC9146106; doi:10.3390/microorganisms10050851)
Supplement: Supplementary file 1 [file microorganisms-10-00851-s001.zip › microorganisms-1679568-supplementary.pdf]

Supplementary information

1. Materials and Methods

1.1. in vitro safety assessment of MJM60396

1.1.1. Hemolytic activity, D-lactate production, biogenic amine production

In vitro safety assessments were carried out on a hemolytic activity, D-lactate production and biogenic amine production.

The hemolytic activity of MJM60396 was assessed using blood agar (Hardy Diagnostics, A10) containing 5% defibrinated sheep blood. MJM60396 was streaked to the blood agar and incubated at 37 ℃ for 24 hours. Hemolytic activity was observed with transmitted light and determined by the photochromic properties around the colonies.

For the evaluation of D-lactate production, candidate strains were cultured in MRS medium at 37°C for 48 hours. The supernatant was analyzed using a D-lactic acid assay kit (Megazyme, K-DATE) according to the manufacturer's protocol.

Bile salt deconjugation was determined by using the standard plate assay method as described, with minor modification [1]. The candidate strains were inoculated to the MRS agar plate supplemented with 0.5% taurodeoxycholic acid sodium salt hydrate (Sigma-Aldrich, Saint Louis, MO, USA), and incubated at 37℃ for 48 hours. Bile salt deconjugation was determined by the visible halo zone and the opaque white precipitate around the colonies.

For the evaluation of biogenic amine production, the decarboxylase medium was supplemented with a different amino acid, as described before [2]. The amino acids used in this study were L-tyrosine, L-histidine, L-ornithine, L-phenylalanine, and L-lysine. Medium without any amino acid was used as a control. The colonies of isolates were streaked on plates with or without amino acid supplied medium (tryptone, 0.5 g; yeast extract, 0.5 g; meat extract, 0.5 g; sodium chloride, 0.25 g; glucose, 0.05 g; tween 80 g, 0.1 g; magnesium sulphate, 0.02 g; manganese sulphate, 0.005 g; ferrous sulphate, 0.004 g; ammonium citrate, 0.2 g; thiamine, 0.001 g; dipotassium phosphate, 0.2 g; calcium carbonate, 0.01 g;

pyridoxal 5-phosphate, 0.005 g; bromocresol purple, 0.006 g; agar, 2 g; pH, 5.3). The plate was incubated in aerobiosis at 37 ºC for 16 to 24 hours and examined for the formation of purple color around the colonies.

1.1.2. Susceptibility of the candidate strains to antibiotics

The minimum inhibitory concentration (MIC) of different antibiotics was determined by using the two-fold broth microdilution method [3]. The antibiotics used for the test were chloramphenicol, ampicillin, tetracycline, gentamycin, kanamycin, streptomycin, erythromycin, clindamycin, and vancomycin, as recommended by European Food Safety Authority (EFSA) [4]. The MIC cutoff values of various antibiotics given by the EFSA for *L. paracasei* were used to determine the antibiotic susceptibility of MJM60396.

1.1.3. Oro-gastrointestinal transit assay

An oro-gastro-intestinal (OGI) transit assay was performed as previously described, with modification [5]. The LAB strains were initially subjected to oral stress by treating bacterial cells (10^9 CFU/mL) in the oral stress solution (NaCl, 6.2 g; KCl, 2.2 g; $CaCl_2$, 0.22 g; $NaHCO_3$, 1.2 g; Lysozyme 0.15 g) at 37 °C for 10 min. After that, the oral stress solution was removed by centrifugation at 1800 × g for 5 min and the cells were subjected to gastric stress by incubating in a gastric solution containing 0.3% pepsin at pH 3 for 1 h. Then, the gastric solution was removed by centrifugation and the cells were incubated for 120 min in the intestinal solution (NaCl, 5 g; KCl, 0.6 g; $CaCl_2$, 0.25 g; pancreatin, 1 g; bile oxgall, 3 g; pH 7). Strains incubated in PBS without stress were used as a control. Cell viability was determined at each step by plating the cells in MRS medium using a serial dilution method and counting the number of colony-forming units (CFU) after 48 h.

1.1.4. Cell culture

The cytotoxicity of candidate strain to HT-29 cells was determined by the MTT assay as described with minor modification [6,7]. The HT-29 cell line was obtained from Korean Collection for Type Cultures (KCTC). The HT-29 cells were cultured in high-glucose minimum essential medium (MEM; Gibco)

supplemented with 20% (v/v) inactivated fetal bovine serum and 100 U of penicillin-streptomycin (Gibco) at 37 °C under 5% CO_2.

1.1.5. Cell adhesion assay

HT-29 cells were seeded at a concentration of 2×10^5 cells/well in a 12-well plate and incubated for 24 hours to get a polarized monolayer and 80% confluence. HT-29 monolayers were washed three times with PBS, and the medium was replaced with antibiotic-free DMEM. Each well was inoculated with bacteria (the final concentration was 1×10^8 CFU/mL) and incubated for 2 hours at 37℃ in 5% CO_2. After incubation, the monolayers were washed three times with PBS, and 200 μL of 1% triton-X 100 (Sigma-Aldrich, Saint Louis, MO, USA) was added. Aliquots of 1 mL homogenate were plated on MRS agar using serial dilution and incubated in anaerobiosis at 37° C for 48 hours. The percentage of bacteria that adhered to the plate was then calculated by dividing the remaining bacteria grown on MRS agar by the initial inoculation bacteria. All the experiments were performed in triplicate.

1.1.6. Antimicrobial assay

The antimicrobial activity of the candidate strain was determined against human, animal, and food-borne pathogens by using the agar well diffusion method [8]. For the antimicrobial test, MJM60396 was incubated in anaerobiosis at 37 ℃ for 24 hours in MRS broth. The pathogenic strains were *Escherichia coli* K99, *Escherichia coli* O1 KCTC 2441, *Escherichia coli* O138, *Escherichia coli* ATCC 25922, *Salmonella enterica* subsp. *enterica* serovar Gallinarum KCTC 2931, *Salmonella enterica* KCTC 2932, *Salmonella enterica* subsp. *enterica* serovar Typhi KCTC 2514, and *Pseudomonas aeruginosa* KCCM 11802. The pathogenic strains (10^6 CFU/mL) were mixed on LB agar. Wells (8 mm diameter) were punched in the plate, and the wells were filled with 100 μL of LAB cell-free culture supernatants of MJM60396 and MJM60662. The plates were incubated in aerobiosis at 37 ℃ for 24 hours, and the antimicrobial activity was determined by measuring the diameters of inhibition zones except for well size (8 mm). All the tests were performed in triplicate.

2. Result

2..1. In vitro safety assessment of MJM60396 of hemolytic activity, OGI transit assay, D-lactate production, biogenic amine production, and susceptibility to antibiotics

Strain MJM60396 was negative for hemolytic activity on blood agar, D-lactate production, bile salt deconjugation, and bioamine production on decarboxylase agar media supplemented with various amino acids (Table 4). Strain MJM60396 was tested for antibiotic susceptibility according to the cutoff value recommended by EFSA (2012) and was found to be susceptible to all antibiotics (Table S1).

The tolerance for OGI transit of MJM60396 and MJM60662 strains was verified for the exposure duration in artificial conditions of oral, gastric, and intestinal transit. MJM60396 decreased 0.2 log unit CFU ($P < 0.1$) in the oral transit assay compared to the initial counts (Table 4). A decrease of 0.67 log unit CFU ($P = 0.0013$) was observed after the oral transit assay for MJM60662. MJM60396 decreased 1.4 log unit CFU ($p < 0.0001$) in the gastric transit assay. A decrease of 0.88 log unit CFU ($p = 0.0002$) was observed after the gastric transit assay for the MJM60662 strain. MJM60396 decreased 1.1 log unit CFU ($p < 0.0001$), and MJM60662 strain did not decrease in the intestinal transit assay. MJM60396 has resistance to lysozyme, but the viability can be seen to decrease as it progresses from gastric to intestinal. MJM60396 decreased 2.7 log unit CFU ($p < 0.0001$) at the end of the OGI transit assay compared to the initial counts. A decrease of 1.6 log unit CFU ($p < 0.0001$) was observed after the OGI transit assay for MJM60662.

2.2. Adherence ability to HT-29 cells

The cell adherent ability was investigated by using human enterocyte-like HT-29 cells. The adherent rates for MJM60396 and MJM60662 were 17.5% and 23.57%, respectively (Table S1). The adhesion rate showed lower adherence of MJM60396 cells as compared to that of MJM60662.

2.3. Analysis of the antibacterial activity of candidate LAB strains

The results of antimicrobial activities showed that MJM60396 strains had antibacterial activity against various enteric pathogens. It strongly inhibited *Escherichia coli* K99, *Escherichia coli* O1 KCTC 2441, and

Salmonella enterica subsp. *enterica* serovar Gallinarum KCTC 2931. MJM60396 showed moderate inhibitory activity against *Escherichia coli* O138, and *Salmonella enterica* subsp. *enterica* serovar Typhi KCTC 2514 (Table S2). The MJM60662 strains also showed strong or moderate antibacterial activity against these pathogens.

Table S1. *In vitro* probiotic characterization and safety assessment of *L. paracasei* MJM60396 and *L. gasseri* MJM60662

Safety test	*L. paracasei* MJM60396	*L. gasseri* MJM60662
Cell viability (%)	170.01	98.47
Cell adhesion rate (%)	17.5	23.57
OGI transit (Log_{10} CFU/mL)		
Initial stress	9.41	8.91
Oral stress	9.22	8.23
Gastric stress	7.86	7.35
Intestinal stress	6.76	7.35
Hemolytic activity	-	-
D-lactate production	-	-
Bile salt deconjugation	-	-
Bioamine production		
L-Phenylalanine	-	-
L-Lysine	-	-
L-Ornithine	-	-
L-Histidine	-	-
L-Tyrosine	-	-
Antibiotics*		
Chloramphenicol	4	4
Ampicillin	1	0.25
Tetracycline	2	2
Vancomycin	128 (n.r)	128 (R)
Gentamycin	32	4
Kanamycin	256 (R)	32 (R)
Streptomycin	64	16
Erythromycin	0.25	0.25
Clindamycin	0.25	4 (R)

R, resistant; -, no activity; n.r, not required

*MIC value (mg/L) for the antibiotics recommended by the European food safety authority (EFSA), 2011

Table S2. Antibacterial activity of candidate LAB strains against enteric pathogens

	Strain	Zone of inhibition (mm)	
		L. paracasei MJM60396	*L. gasseri* MJM60662
1	*Escherichia coli* K99	10	8
2	*Escherichia coli* O1 KCTC 2441	8	6
3	*Escherichia coli* O138	6	6
4	*Escherichia coli* ATCC 25922	8	8
5	*Salmonella enterica* subsp. *enterica* serovar Gallinarum KCTC 2931	8	6
6	*Salmonella enterica* KCTC 2932	2	4
7	*Salmonella enterica* subsp. *enterica* serovar Typhi KCTC 2514	6	6

References

1. Hernández-Gómez, J.G.; López-Bonilla, A.; Trejo-Tapia, G.; Ávila-Reyes, S.V.; Jiménez-Aparicio, A.R.; Hernández-Sánchez, H. In Vitro Bile Salt Hydrolase (BSH) Activity Screening of Different Probiotic Microorganisms. *Foods* **2021**, *10*, 674, doi:10.3390/foods10030674.
2. Palaniyandi, S.A.; Damodharan, K.; Suh, J.-W.; Yang, S.H. In Vitro Characterization of Lactobacillus Plantarum Strains with Inhibitory Activity on Enteropathogens for Use as Potential Animal Probiotics. *Indian J Microbiol* **2017**, *57*, 201–210, doi:10.1007/s12088-017-0646-4.
3. Wiegand, I.; Hilpert, K.; Hancock, R.E.W. Agar and Broth Dilution Methods to Determine the Minimal Inhibitory Concentration (MIC) of Antimicrobial Substances. *Nat Protoc* **2008**, *3*, 163–175, doi:10.1038/nprot.2007.521.
4. Guidance on the Assessment of Bacterial Susceptibility to Antimicrobials of Human and Veterinary Importance. *EFSA Journal* **2012**, *10*, 2740, doi:10.2903/j.efsa.2012.2740.
5. Bove, P.; Gallone, A.; Russo, P.; Capozzi, V.; Albenzio, M.; Spano, G.; Fiocco, D. Probiotic Features of Lactobacillus Plantarum Mutant Strains. *Appl Microbiol Biotechnol* **2012**, *96*, 431–441, doi:10.1007/s00253-012-4031-2.
6. Damodharan, K.; Lee, Y.S.; Palaniyandi, S.; Yang, S.H.; Suh, J.-W. Preliminary Probiotic and Technological Characterization of Pediococcus Pentosaceus Strain KID7 and in Vivo Assessment of Its Cholesterol-Lowering Activity. *Frontiers in Microbiology* **2015**, *6*, 768, doi:10.3389/fmicb.2015.00768.
7. Soncin, F.; Shapiro, R.; Fett, J.W. A Cell-Surface Proteoglycan Mediates Human Adenocarcinoma HT-29 Cell Adhesion to Human Angiogenin. *Journal of Biological Chemistry* **1994**, *269*, 8999–9005, doi:10.1016/S0021-9258(17)37067-9.
8. Tagg, J.R.; McGiven, A.R. Assay System for Bacteriocins. *Appl Microbiol* **1971**, *21*, 943.
